# The Future of the HIV Response: Sustaining Progress in an era of Political Uncertainty and Scientific Opportunity

**DOI:** 10.1111/aji.70287

**Published:** 2026-07-11

**Authors:** Mitchell Warren

**Affiliations:** ^1^ AIDS Vaccine Advocacy Coalition (AVAC) New York City USA

**Keywords:** global health, HIV, implementation, innovation, NIH, PEPFAR, PrEP

## Abstract

The global HIV response is at a pivotal juncture. The field is experiencing a paradox: rapid scientific innovation alongside increasing fragility in financing, political commitment, and implementation systems. Significant progress has been made in expanding access to HIV treatment and prevention, but major gaps in coverage and equity existed previously, that were further exacerbated and destabilized by changes in US government support in January 2025. This commentary examines the implications of this divergence and argues that the future of the HIV response depends less on new biomedical discoveries than on the capacity to deliver existing and emerging options sustainably and with speed, scale and equity. Drawing on recent advances in HIV prevention and treatment, as well as massive disruptions and cuts in both global health programs, including PEPFAR, and biomedical research funding under the new US Presidential Administration, the paper identifies key priorities for the next phase of the response, including strengthening health systems, ensuring equitable access, re‐centering community leadership, and securing long‐term political and financial commitments. Without decisive action, the gains of the past four decades risk stagnation or, worse, actual reversal; with it, the goal of ending the HIV epidemic may still be achievable.

## 1 Introduction

More than forty years after the identification of HIV, the global response stands at a moment of both extraordinary promise and profound uncertainty. Scientific advances have transformed HIV from a fatal diagnosis into a manageable chronic condition and have introduced highly effective prevention strategies capable of dramatically reducing new infections. Yet, the infrastructure required to translate these advances into population‐level impact is increasingly fragile.

This paradox—innovation without implementation—defines the current phase of the HIV response. On the one hand, the pipeline of new prevention and treatment continues to expand, including long‐acting injectable agents. On the other hand, funding instability, shifting political priorities, and systemic inequities threaten to undermine progress.

This moment demands a reframing of priorities. The central challenge is no longer the absence of effective tools, but the failure to deliver them equitably and at scale. The trajectory of the HIV epidemic will not be determined primarily by scientific breakthroughs alone, but by the social, political, and economic systems that govern their use. This commentary examines the structural challenges facing the HIV response and outlines a framework for regaining and then sustaining progress in an increasingly complex global health landscape.

## 2 The Evolution of the HIV Response

The history of the HIV response is often characterized by three overlapping phases: crisis, scale‐up, and optimization.

## 3 From Crisis to Control to Instability

In the early years of the epidemic, the absence of effective treatment and the rapid spread of HIV created a global health emergency. The development of combination antiretroviral therapy (ART) in the mid‐1990s marked a turning point, transforming HIV into a manageable chronic disease for those with access to treatment, which initially was only in wealth countries. It, unfortunately, left millions of people in low‐ and middle‐income countries and from marginalized and stigmatized communities without substantial access until the 2000s.

The subsequent scale‐up of ART, supported by the major global initiatives launched in the early 2000s—the U.S. President's Emergency Plan for AIDS Relief (PEPFAR) and the Global Fund to Fight AIDS, Tuberculosis and Malaria—represents one of the most ambitious and successful public health efforts in history. By the early 2020s, tens of millions of people were receiving treatment, and AIDS‐related mortality had declined substantially [1].

In 2020, the Member States of the United Nations adopted a target of ending HIV/AIDS as a public threat by 2030. While there was progress towards expanding treatment access, the prevention targets were lagging behind in 2024, even before the massive disruptions caused by the new US Presidential Administration beginning in January 2025. The US Government is the largest international funder of the HIV response, accounting for 75% of support for HIV, and the global response immediately entered an era of instability that continues today [2].

## 4 The Prevention Revolution

The scientific evidence of voluntary medical male circumcision (VMMC) in 2007 [3, 4, 5] and then pre‐exposure prophylaxis (PrEP) beginning in 2010 [6, 7] further expanded the range of HIV prevention options. VMMC is the only “one and done” HIV prevention option, conveying strong protective benefit. Daily oral PrEP demonstrated high efficacy when taken consistently, and subsequent innovations have sought to address adherence challenges through long‐acting formulations.

These advances have shifted the paradigm from reactive treatment to proactive prevention. But the development of these new, potent biomedical options has not been sufficient; the effectiveness of translating these options into actual choices that people will use depends on their accessibility, uptake and consistent use, provider and policy maker support, and demand creation for HIV prevention generally.

Although first approved in 2012, oral PrEP failed to deliver impact for the first decade, massively missing the UNAIDS target of three million PrEP users by 2020. Challenges related to individual reactions to the product among both potential users and providers as well as motivation to seek HIV prevention generally; low community awareness of PrEP; health system bottlenecks; and underlying structural barriers all contributed to this slower‐than‐hoped‐for introduction [8]. Some of these factors were consistent with the early introduction of VMMC [9].

**FIGURE 1:**
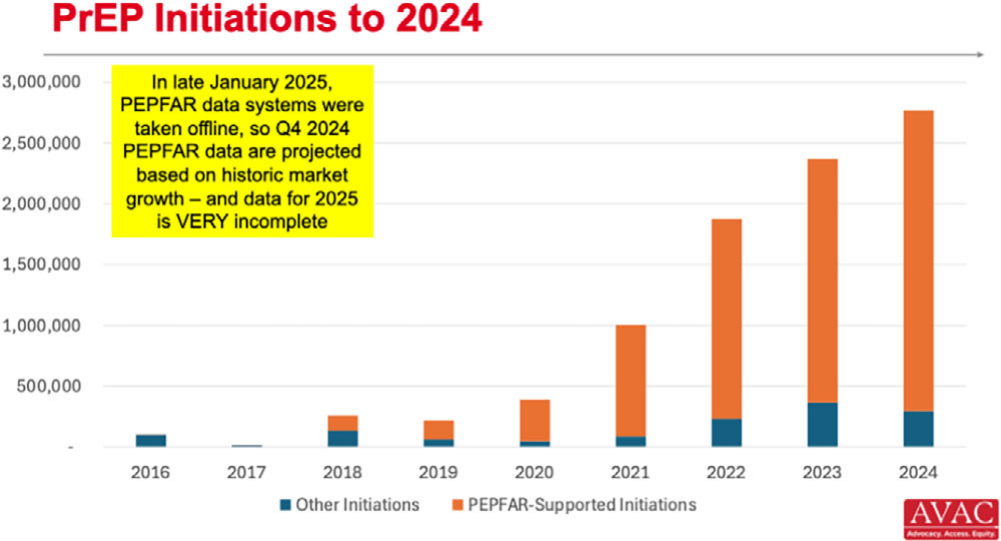
PrEP initiations to 2024.

Oral PrEP uptake did begin increasing in 2021, due largely to significant investments from PEPFAR. The PrEP market grew substantially over the next four years (Figure 1). Unfortunately, in late January 2025, PEPFAR drastically restricted access to PrEP and primary HIV prevention programs [10] and data systems were taken offline, so there is little known about the PrEP market in 2025.

## 5 Entering the Implementation era

The current phase of the HIV response can best be understood as the “implementation era”. The pace of scientific innovation in HIV prevention and treatment has accelerated in recent years, offering new opportunities to reduce transmission and improve outcomes. The key challenge is ensuring the equitable delivery of effective interventions—with speed and at scale. This requires a focus on system design, from efficacy to access, and from global targets to local realities, while still maintaining a robust pipeline of additional options that are still required to fill the gap, including an HIV vaccine and an HIV cure or remission strategy.

Long‐acting injectable agents represent one of the most significant advances in HIV prevention. Cabotegravir, administered every two months, has demonstrated superior efficacy compared to daily oral PrEP in clinical trials [11, 12]. Lenacapavir, a capsid inhibitor with a dosing interval of six months, offers the potential for even greater convenience and adherence [13, 14]. Additional innovations—such as a monthly oral PrEP candidate in efficacy trials and expanded delivery platforms, focusing on self‐care and integrating artificial intelligence—signal a future defined by choice and personalization in prevention.

These approaches address a critical limitation of treatment and earlier prevention strategies: the need for consistent daily adherence. By reducing the frequency of dosing, long‐acting agents may improve uptake and persistence, particularly among populations facing structural barriers to care.

## 6 Treatment Innovations

Advances in treatment, including long‐acting ART formulations and simplified regimens, have improved quality of life for people living with HIV. These innovations also have implications for prevention, as effective treatment reduces viral load and eliminates the risk of sexual transmission. Beginning with the groundbreaking HPTN 052 study results in 2011 [15] that provided clear evidence that treatment was also prevention, advocates, researchers and policy makers began a new focus, campaigns and clinical guidance about the power of “U = U”, or Undetectable = Untransmittable [16].

## 7 The Implementation Gap

Despite these advances, the global HIV response faces a persistent and widening gap between what is scientifically possible and what is programmatically achieved.

The uptake of PrEP and other prevention options has been uneven, constrained by cost, infrastructure requirements, and limited health system readiness, with significant disparities across regions and populations. Similarly, access to the first‐generation of injectable HIV treatment has not reached low‐ and middle‐income countries [17].

Factors contributing to low uptake include:

- Cost and affordability
- Limited health system capacity
- Stigma and discrimination
- Lack of awareness and demand

These barriers are particularly pronounced among key populations, including men who have sex with men, sex workers, transgender individuals, and people who inject drugs, and among adolescent girls and young women.

## 8 Health System Constraints

Delivering long‐acting technologies requires robust health systems capable of supporting regular clinical visits, cold chain logistics, and trained personnel. In many settings, these requirements exceed current capacity. Without significant investment in health system strengthening, new technologies may exacerbate existing inequities rather than reduce them.

## 9 A Fragile Global Health Architecture

The global HIV response has historically relied on a small number of major donors, particularly PEPFAR and the Global Fund. While these programs have been highly effective, their dominance creates vulnerability, that became more apparent than ever in the midst of the new US Presidential Administration in January 2025.

**FIGURE 2:**
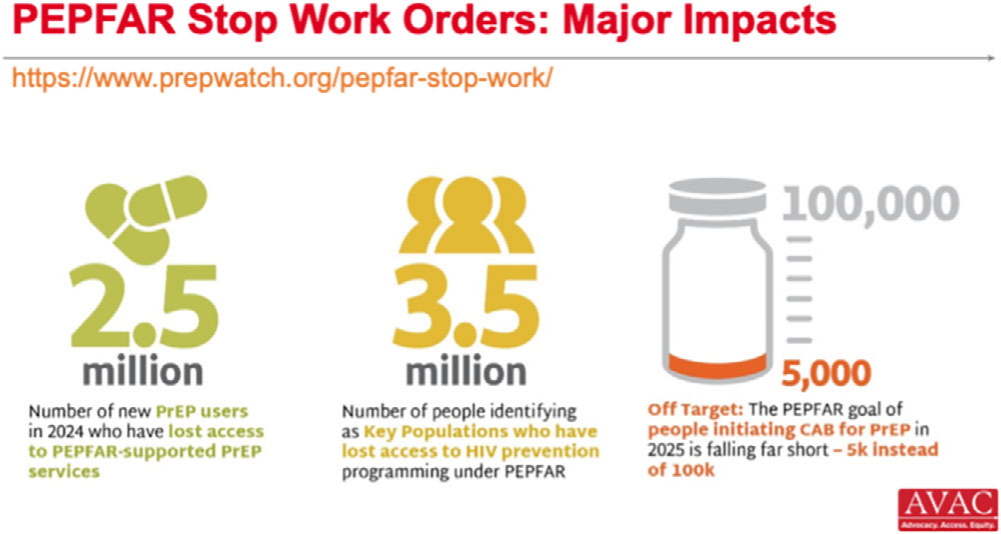
Impacts of PEPFAR stop work orders on prevention.

**FIGURE 3:**
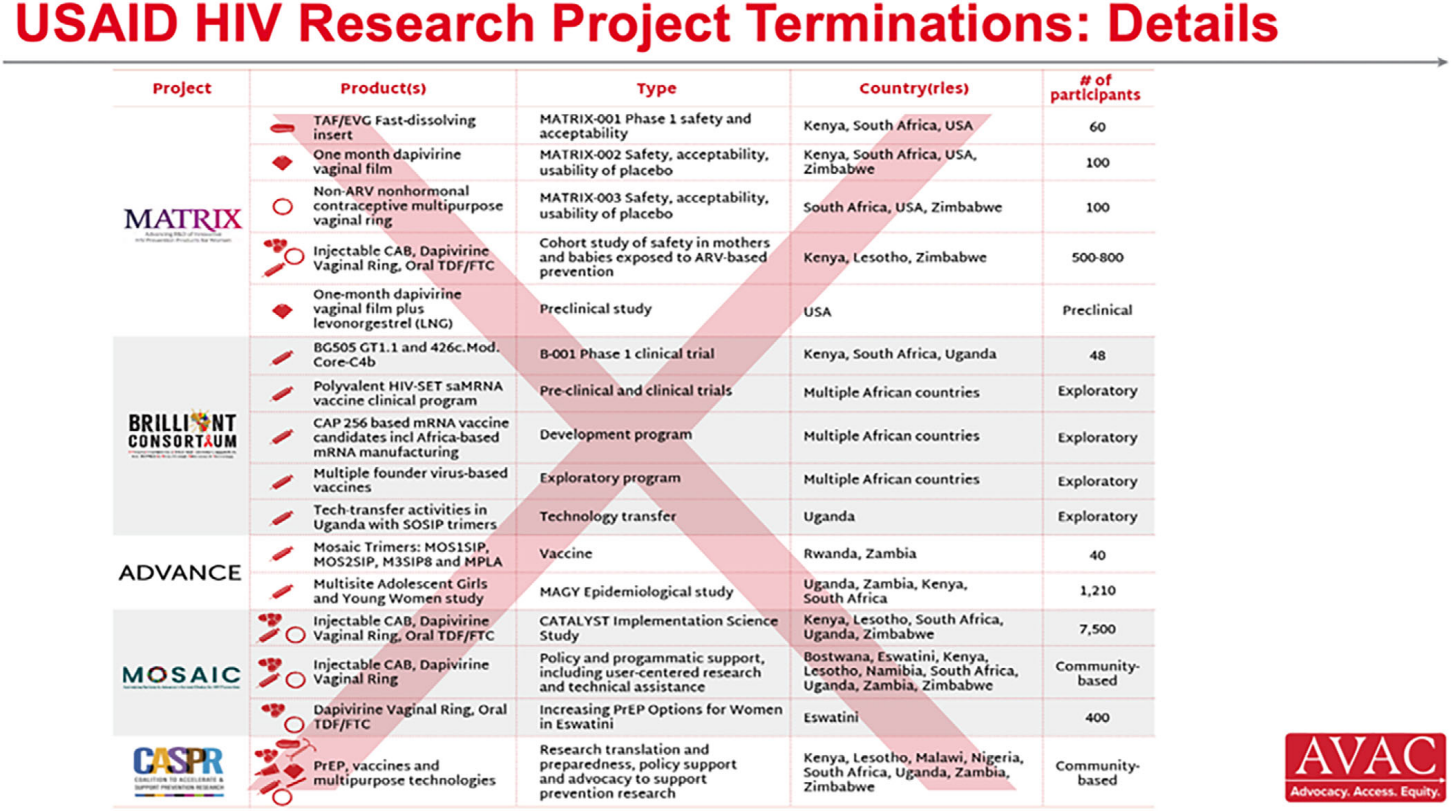
Terminated USAID‐funded HIV research projects.

PEPFAR—long considered the backbone of global HIV treatment and prevention—faces uncertainty, delays, and partial reductions. Disruptions in PEPFAR funding had immediate implications beginning in February 2025, especially with respect to major cuts in primary prevention, PrEP and key population programming (Figure 2). These cuts and termination also undermined the introduction of injectable cabotegravir for PrEP, research pipelines, including clinical trials for new PrEP options and vaccines, trust and confidence at the community level and foundational links between HIV testing and treatment (Figure 3).

**FIGURE 4:**
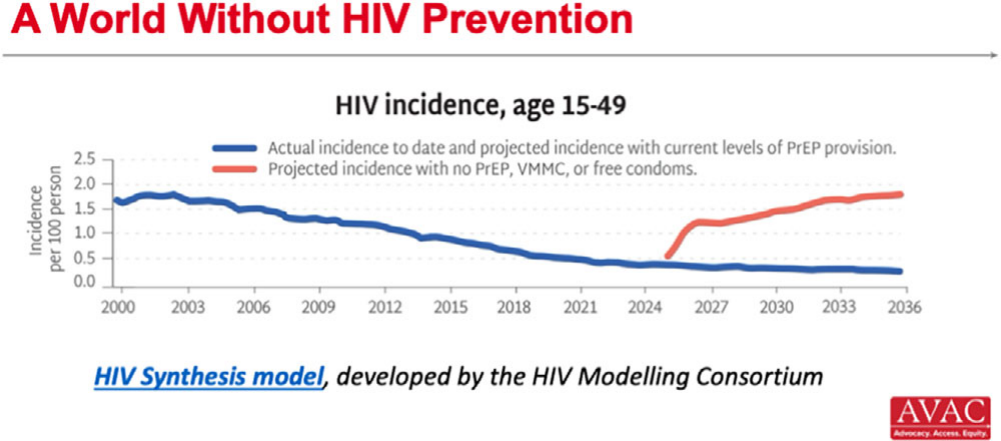
Model projections of the epidemic without HIV prevention.

The failure to re‐start and re‐invest in HIV testing, treatment, primary prevention and key population programming will have severe consequences. The HIV Synthesis model developed by the HIV Modelling Consortium predicts that without rapid reversal of the current situation could lead to HIV incidence returning to rates last seen in the early 2000s (Figure 4). In this analysis, after years of progress in reducing HIV incidence, without the restart of investments in HIV testing, VMMC, PrEP and condoms within the next three years, HIV incidence among 15‐ and 49‐year‐olds in East and Southern Africa will return to rates in 2001 – the time before PEPFAR or Global Fund existed and made HIV treatment a reality for millions of people.

This fragility exposes a deeper structural problem: the HIV response has been overly dependent on a small number of donors and centralized funding streams. When those falter, entire national programs—from prevention delivery to clinical trials—are jeopardized. The future response must, therefore, prioritize resilience, including diversified financing, stronger domestic investment, and regionally‐led governance.

To enhance resilience, the HIV response must diversify its funding base. This includes:

- Increasing domestic investments in health in all countries, particularly those that have previously relied on PEPFAR and the Global Fund.
- Engaging new bilateral and multilateral donors.
- Exploring innovative financing mechanisms.

Domestic resource mobilization is particularly important for ensuring long‐term sustainability and local ownership, and is a core tenant of the new “America First” Global Health Strategy and the current focus on US‐led negotiations for new, bilateral memoranda of understanding [18].

## 10 Sustaining the Research Enterprise

While recent innovations have energized the field, the HIV/AIDS response has always been driven by biomedical research, and there remains a robust and urgent research agenda, including the development of additional prevention and treatment options, vaccines and a cure. While scaling up existing treatment and prevention options, including new innovations like lenacapavir, today, they do not eliminate the need for sustaining research endeavors for the tools needed to durably end the epidemic. Unfortunately, in addition to recent disruptions of US foreign assistance programs described above, the current US Administration has similarly disrupted international HIV research, especially at the National Institutes of Health (NIH).

Through various Executive Orders and administration actions, there have been unprecedented funding terminations and disruptions in basic science, clinical trials, international sub‐awards and the scientific review processes [19].

**FIGURE 5:**
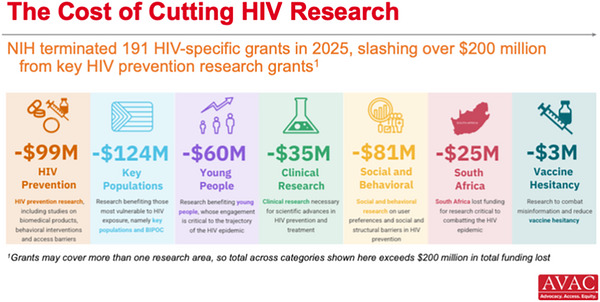
The cost of cutting research investments.

Within the first half of 2025, the NIH terminated 191 HIV‐specific grants, slashing over $200 million from key HIV research grants resulting in significant potential implications for HIV prevention, vaccine, treatment, cure and social and behavioral science (Figure 5). These research disruptions may not disrupt health care delivery immediately in the way PEPFAR cuts have, but they do jeopardize the development of future innovations.

The current excitement for lenacapavir is a useful case study: it took nearly twenty years of NIH‐supported basic research in academia before Gilead Sciences invested in the development of the actual product, which was then studied in clinical trials that were conducted globally in clinical trial sites that were developed over many years with NIH investments [20]. Without a robust research enterprise, there may not be the “next lenacapvir” for HIV or for other chronic, infectious and/or non‐communicable diseases.

## 11 Equity and Access: The Central Challenge

Equity has been a defining principle of the HIV response, yet disparities persist. The early years of ART rollout were marked by significant delays in access for low‐ and middle‐income countries. Over time, advocacy, generic manufacturing, and international financing mechanisms helped to close this gap. Now, these lessons must be adapted and applied strategically to new prevention technologies. Access, not innovation, is now a primary bottleneck, and delayed access is not an inevitable outcome; it is a policy choice.

Ensuring affordability will require coordinated efforts to shape markets, and delivery systems must be built now so that when generics and lower‐cost options emerge, scale‐up can happen rapidly. Without this groundwork, new technologies risk repeating the inequities seen in earlier HIV treatment and PrEP rollouts. The future HIV response must shift from a product‐centric model to a delivery‐centric model, focusing on:

- Health system strengthening
- Community‐led distribution
- Pricing and market‐shaping strategies, including early planning for generic manufacturing and licensing and pricing agreements
- Integration with broader primary care services
- Strengthening supply chains and service delivery platforms
- Leveraging pooled procurement mechanisms

These strategies have been successful in the past and remain essential for future scale‐up, and failure to address these issues risks perpetuating global inequities and limiting the population‐level impact of new innovations [21].

Moreover, equity is not an adjunct goal; it is the central determinant of whether biomedical advances translate into population‐level impact.

## 12 Re‐Centering Civil Society Leadership and Local Community Engagement

Community engagement, community‐based organizations and robust accountability mechanisms have been a cornerstone of successful HIV responses, from early activism to current service delivery and monitoring. Civil society involvement and leadership enhances trust, improves uptake of products and services, and ensures that interventions are responsive to local needs.

However, civic space is shrinking, and community organizations are increasingly underfunded and excluded from decision‐making processes, especially in the wake of changes in the PEPFAR program and the new US global health strategy. One of PEPFAR's hallmarks over its first 20 years has been in the tripartite relationship among the US and host governments and civil society [22]. But the new “America First Global Health Strategy” launched in September 2025 focuses on transactional government‐to‐government relationships [23]. This undermines both effectiveness and equity, particularly for key populations who remain disproportionately affected by HIV.

Re‐centering communities is essential for both ethical and practical reasons. Community‐led interventions have been shown to improve uptake, adherence, and retention in care, particularly among key populations [24]. Moreover, addressing structural barriers such as stigma, discrimination, and criminalization requires sustained advocacy and grassroots leadership. The future HIV response must therefore prioritize community leadership as a core component, rather than a supplementary element.

## 13 Addressing Structural Barriers

Ending the HIV epidemic requires addressing the structural determinants of risk, including:

- Stigma and discrimination
- Criminalization of key populations
- Gender inequality
- Economic marginalization

These factors cannot be addressed through biomedical interventions alone. They require sustained political and social change.

## 14 Integration and Health System Strengthening

Vertical HIV programs that often rely on dedicated funding, distinct staffing, systems and sometimes even facilities, have achieved remarkable success, but their sustainability is increasingly questioned. Integrating HIV services into broader health systems can enhance efficiency and resilience [25]. Going forward, integration of HIV programs within broader health systems—particularly within the framework of universal health coverage—can enhance efficiency, resilience, and continuity of care. This approach also facilitates the delivery of HIV services alongside other essential health interventions, such as sexual and reproductive health, tuberculosis treatment and prevention, and primary care, thereby improving overall health outcomes.

## 15 Political Will and Global Governance

From the beginning in the 1980s, political commitment has been a driving force behind the HIV response. Advocacy by civil society, affected communities, and global organizations has secured funding, shaped policies, and held governments accountable. Now, renewed advocacy is essential to sustain progress, particularly in the face of competing global priorities.

Effective global governance is critical for coordinating efforts, sharing knowledge, and mobilizing resources. Institutions such as UNAIDS and WHO have played key roles, but their effectiveness depends on the support—both financial and political—of member states. Renewed advocacy will be essential to maintain HIV as a global priority. This includes holding governments and donors accountable, as well as mobilizing new constituencies to support the response.

## 16 The Risk of Complacency

One of the most significant threats to the HIV response is complacency. Declining visibility of the epidemic in some regions, combined with competing global health challenges, has reduced the sense of urgency. Without sustained effort, the epidemic will persist, and gains may be reversed.

## 17 A Framework for the Future

The future HIV response must now focus on five key priorities:

1. **Scaling Access to Existing Options**: Prioritize the rapid and equitable rollout of proven interventions.
2. **Strengthening Health Systems**: Invest in infrastructure, workforce, and service delivery platforms.
3. **Ensuring Sustainable Financing**: Diversify funding sources and increase domestic investment.
4. **Maintaining Innovation and Investments in R&D**: While multiple options exist, continued research is needed to optimize delivery, develop vaccines and a cure, and address emerging challenges.
5. **Re‐centering Communities**: Empower community organizations and address structural barriers.

## Conclusion

The global HIV response stands at a crossroads. A broader array of options to significantly reduce new infections and improve outcomes now exists, and additional innovations are on the horizon. Yet the systems required to deliver these tools are under enormous strain. The future of the HIV response will be determined not by science alone, but by the political, economic, and social contexts in which that science is deployed.

In this sense, the next decade of the HIV response is not primarily a scientific endeavor; it is a test of global solidarity, political courage, and commitment to equity. Without these, even the most extraordinary innovations will fall short. With them, the long‐envisioned goal of ending the HIV epidemic may finally come within reach.

## Ethics Statement

The author confirms that the ethical policies of the journal, as noted on the journal's author guidelines page, have been adhered to. No ethical approval was required as this is a review article with no original research data.

## Conflicts of Interest

The author leads AVAC, which has taken policy positions advocating for funding of PEPFAR and the NIH, international research and global health equity. AVAC is also the lead plaintiff in *AVAC v. The US Department of State*, a case still active in the US courts that seeks emergency relief from a 2025 Executive Order that froze foreign assistance funding.

## Data Availability

Data sharing not applicable to this article as no datasets were generated or analyzed during the current study.
